# Supplementary material for: Lifespan prolonging mechanisms and insulin upregulation without fat accumulation in long-lived reproductives of a higher termite
Source: Commun Biol. 2022 Jan 13;5:44. doi: 10.1038/s42003-021-02974-6 (PMC8758687; doi:10.1038/s42003-021-02974-6)
Supplement: Supplementary file 2 — Supplementary Information [file 42003_2021_2974_MOESM2_ESM.pdf]

SUPPLEMENTARY INFORMATION

Lifespan prolonging mechanisms and insulin upregulation without fat accumulation in long-lived reproductives of a higher termite

Sarah Séité[1,2#], Mark C. Harrison[3#], David Sillam-Dussès[4], Roland Lupoli[1,2], Tom J. M. Van Dooren[5,6], Alain Robert[4], Laure-Anne Poissonnier[7], Arnaud Lemainque[8], David Renault[9,10], Sébastien Acket[11], Muriel Andrieu[12], José Viscarra[13], Hei Sook Sul[13], Z. Wilhelm de Beer[7], Erich Bornberg-Bauer[3] and Mireille Vasseur-Cognet[1,2,14*]

1- UMR IRD 242, UPEC, CNRS 7618, UPMC 113, INRAe 1392, Paris 7 113, Institute of Ecology and Environmental Sciences of Paris, Bondy, France.
2- University of Paris-Est, Créteil, France.
3- Institute for Evolution and Biodiversity, University of Münster, Münster, Germany.
4- University Sorbonne Paris Nord, Laboratory of Experimental and Comparative Ethology UR4443, Villetaneuse, France.
5- UMR UPMC 113, IRD 242, UPEC, CNRS 7618, INRA 1392, PARIS 7 113, Institute of Ecology and Environmental Sciences of Paris, Paris, France.
6- Naturalis Biodiversity Center, Leiden The Netherlands.
7- Department of Biochemistry, Genetics and Microbiology, Forestry and Agricultural Biotechnology Institute, University of Pretoria, Pretoria, South Africa.
8- Genoscope, François-Jacob Institute of Biology, Alternative Energies and Atomic Energy Commission, University of Paris-Saclay, Evry, France.
9- University of Rennes, CNRS, ECOBIO (Ecosystems, biodiversity, evolution) - UMR 6553, Rennes, France.
10- University Institute of France, Paris, France
11- University of Technology of Compiègne, UPJV, UMR CNRS 7025, Enzyme and Cell Engineering, Royallieu research Center, Compiègne, France.
12- Cochin Institute, UMR INSERM U1016, CNRS 8104, University of Paris Descartes, CYBIO Platform, Paris, France.
13- Department of Nutritional Sciences and Toxicology, University of California, Berkeley, USA
14- INSERM, Paris, France

* Correspondence and requests for materials should be addressed to MVC
email: mireille.vasseur@inserm.fr
https://orcid.org/0000-0001-6963-4114
#These authors contributed equally

This PDF file includes:

Supplementary Figures 1 to 8
Supplementary Table 1 to 8

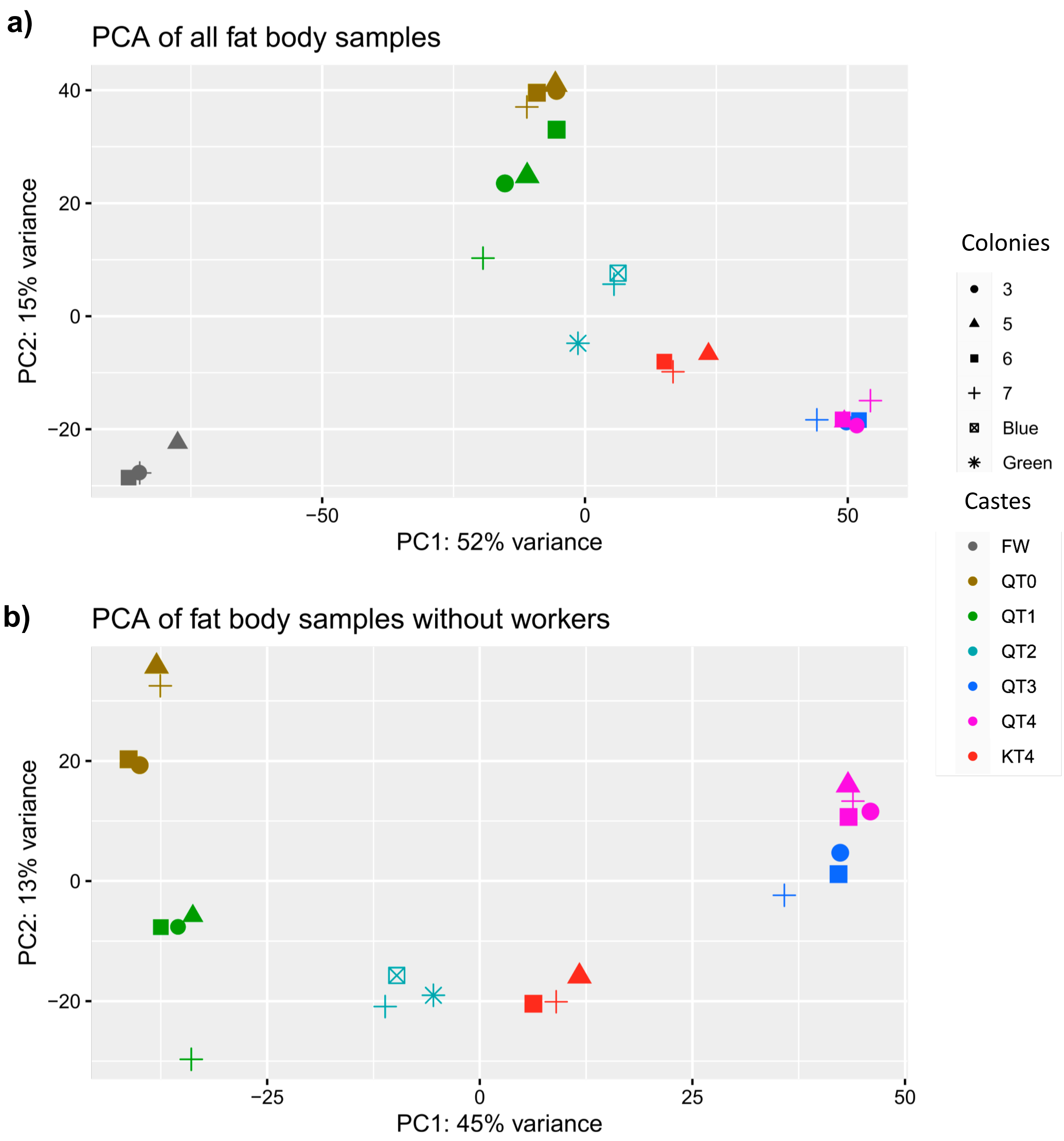

Supplementary Figure 1: Principal component analyses (PCAs) of gene expression across FW, QT0, QT1, QT2, QT3, QT4 and KT4 samples. Scores for the two main PCs are shown for all data (**a**) and among reproductives (i.e., without FW; **b**).

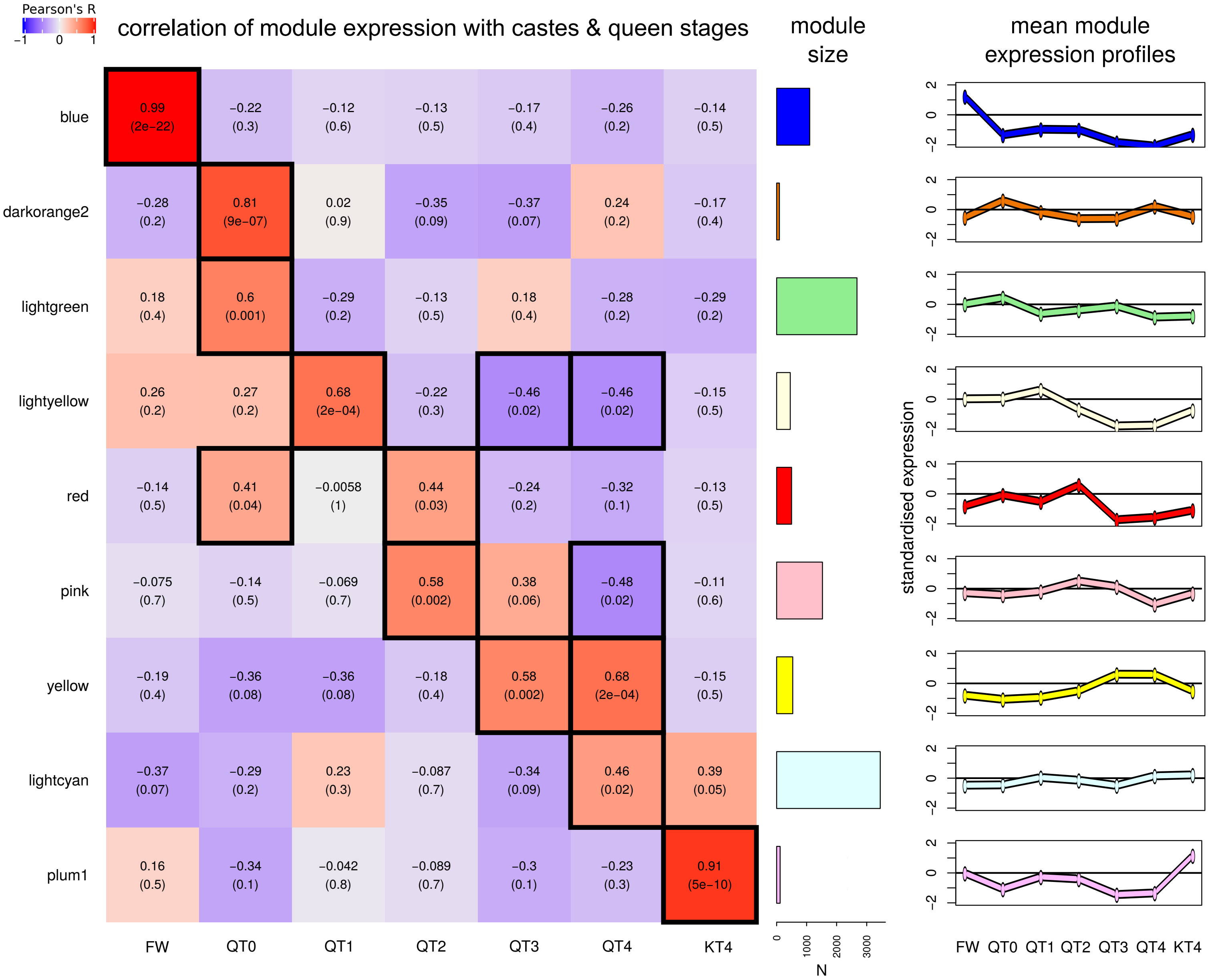

Supplementary Figure 2: Correlation of Weighted Gene Co-expression Network Analysis (WGCNA) modules with caste expression. In the left-hand heatmap correlation coefficients and p-values are shown, relating gene expression (first major axis of the expression matrix) of each module to each caste and queen stage (columns). Positive correlations are red and negative correlations are blue; significant correlation values ($p < 0.05$) have a black border. Module size is also shown, reflecting the number of genes assigned to each module. Module expression profiles show mean expression of each module within each caste and queen stage.

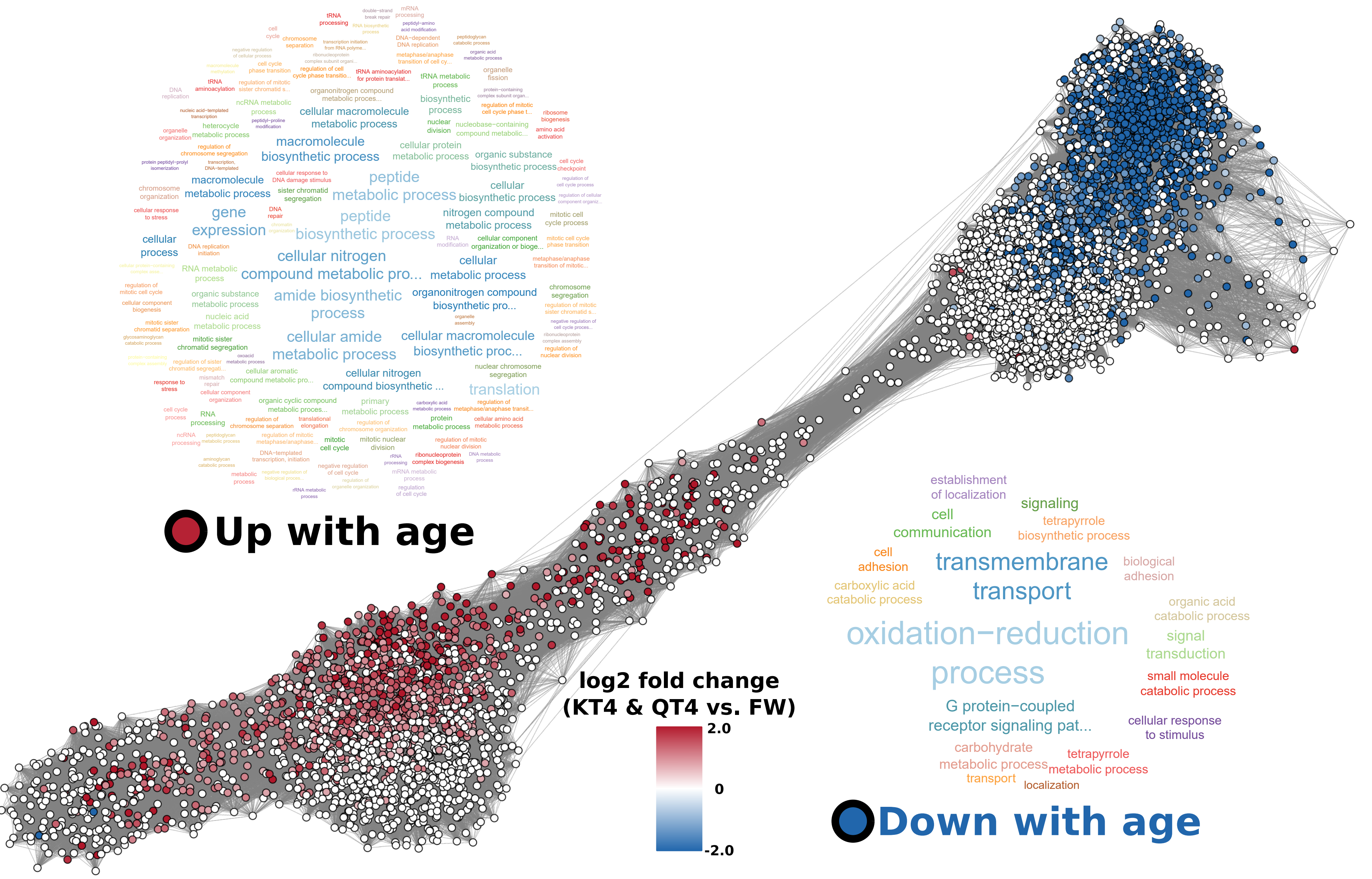

Supplementary Figure 3: Differences between female workers (FW) and reproductives (QT4 and KT4) in gene expression within the GCN. Genes which are significantly up- or down-regulated in both KT4 and QT4 compared to FW are colored by their relative change in expression (log 2-fold change). Significantly enriched GO-terms in each gene group are depicted as tag clouds.

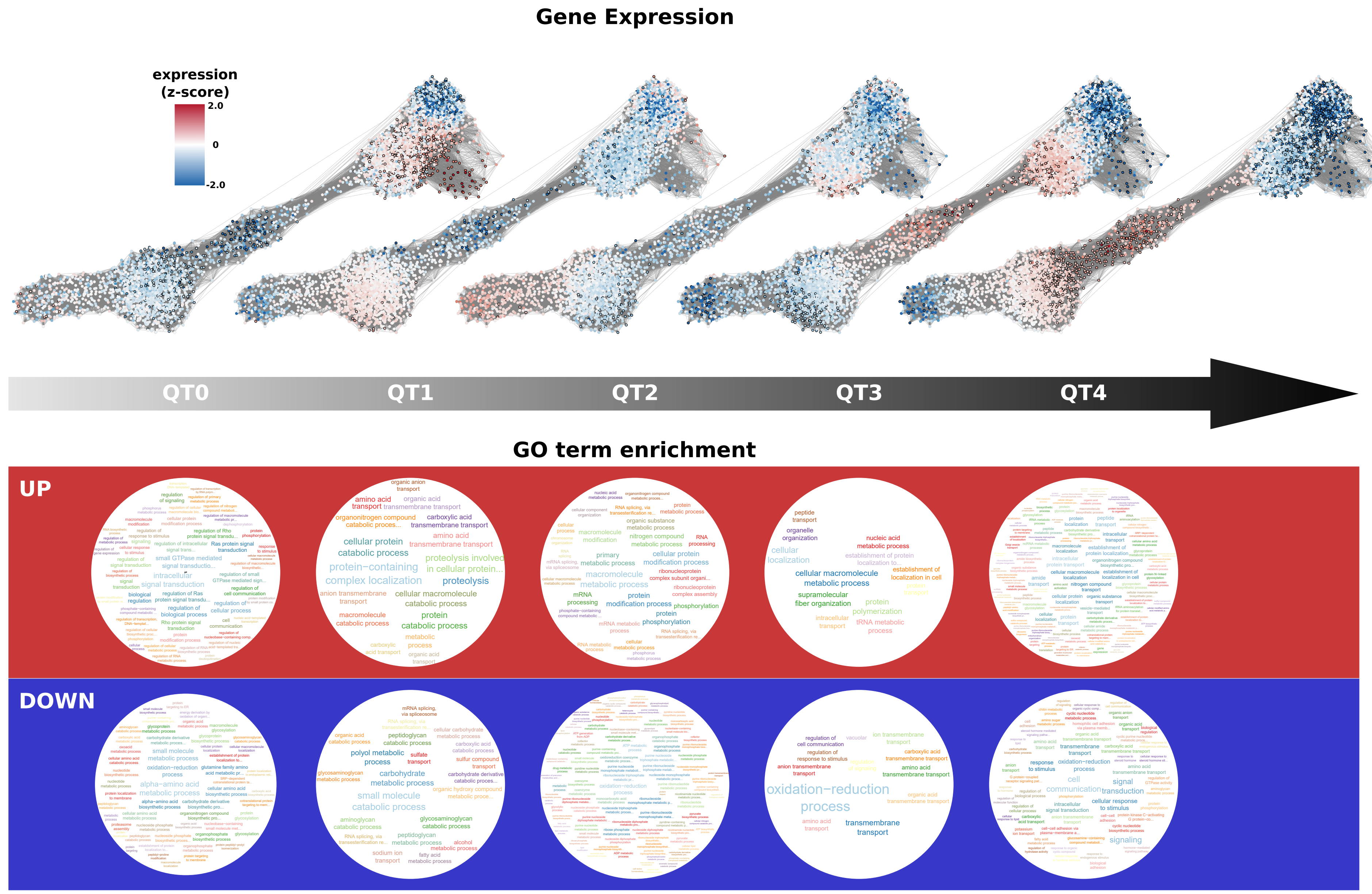

Supplementary Figure 4: Weighted Gene Co-Expression Network (WGCN) during adult queen maturation. Changes of gene expression within the gene co-expression network (GCN) with queen maturation. Node color represents the standardized gene expression within each queen stage (low=blue: high=red). Genes that are significantly up- or down-regulated at each queen stage have a strong, black border. Enriched GO-terms within each group of up- or down-regulated genes are displayed as tag clouds below each network.

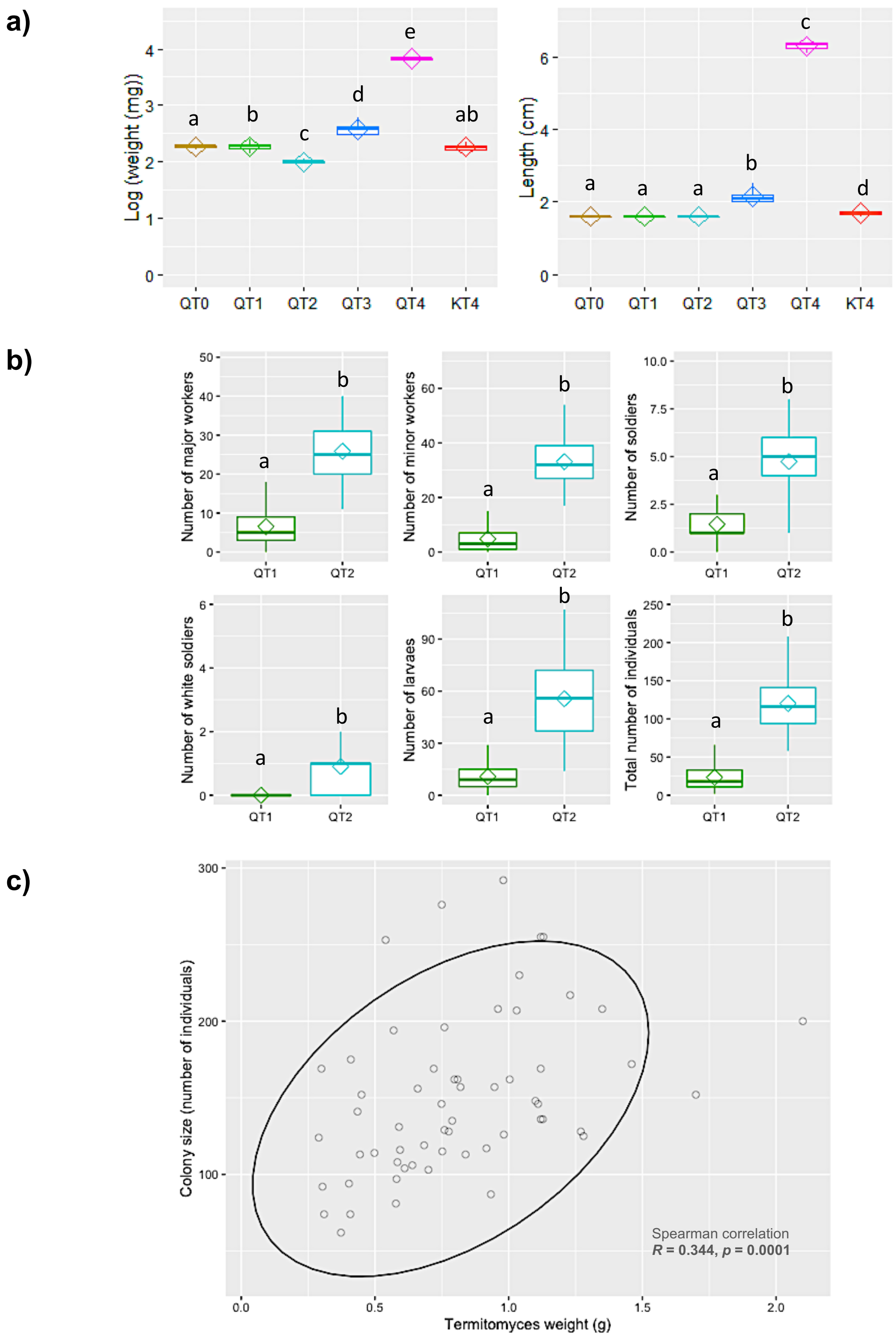

Supplementary Figure 5: Colony foundation of *Macrotermes natalensis*. (a) Log of weight (left) and length (right) of the *M. natalensis* queens (Q) and kings (K) measured at the different sampling times given in Figure 1. Weight and length were measured on 20 QT0, 20 QT1, 20 QT2, 3 QT3, 8 QT4 and 6 KT4. A box consists of upper and lower hinges and a center line corresponding to the 25th percentile, the 75th percentile and the median, respectively. Different letters indicate significantly different values according to a Kruskal-Wallis test followed by Dunn test. Statistical differences are given for *p-value* < 0.05. **(b)** Number of larvae, small workers (female), large workers (male), presoldiers, soldiers and total individuals (sum of all the castes) at two different sampling times given in Figure 1. Different letters indicate significantly different values according to a t-test. Statistical differences are given for *p-value* < 0.05. **(c)** Positive correlation between the weight of the fungus Termitomyces and the number of individuals in colonies sampled at T2. R corresponds to the Spearman coefficient and *p* is the corresponding *p-value*.

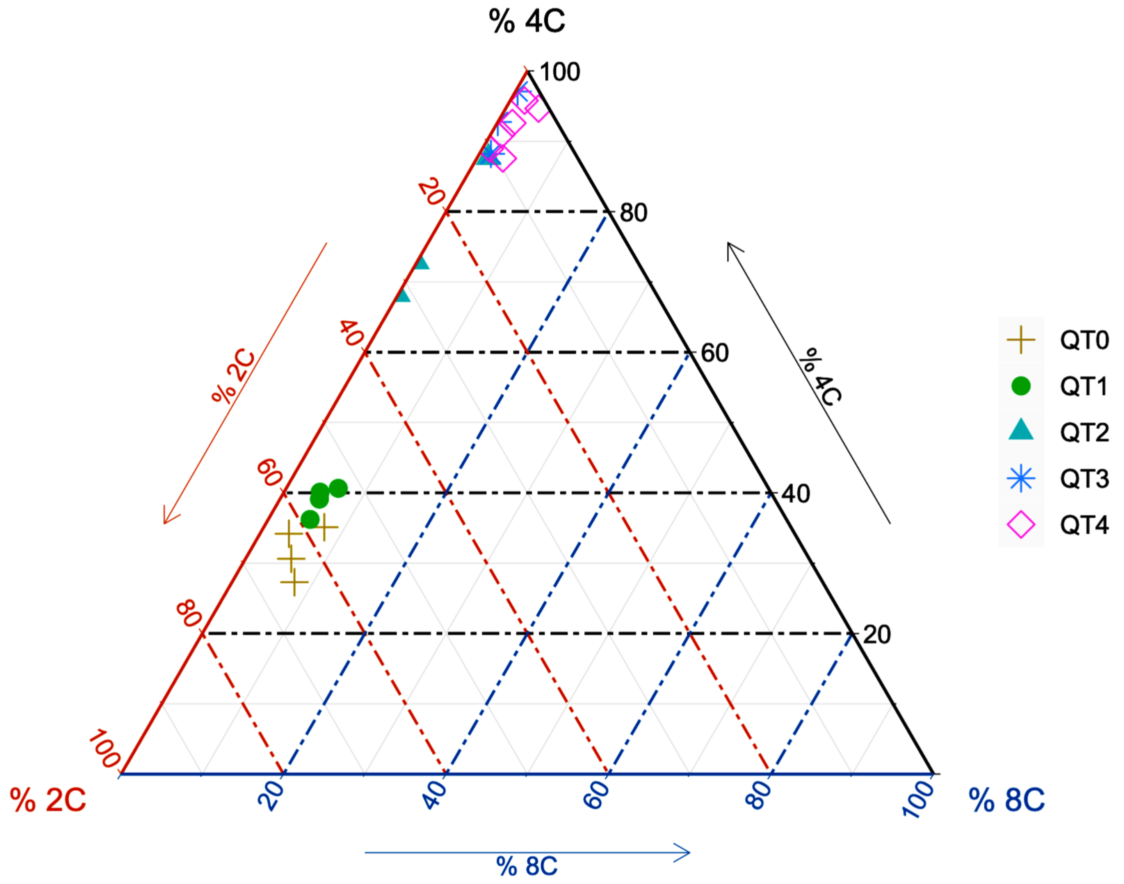

	Queen maturation stages				
**DNA content**	QT0	QT1	QT2	QT3	QT4
**2C (%)**	62.1	55.8	23.3	6.8	5.9
**4C (%)**	31.8	39.0	76.2	92.7	91.4
**8C (%)**	6.1	5.2	0.5	0.6	2.6

Supplementary Figure 6: Changes in DNA content of fat body cells during adult queen maturation. Percentages of nuclear 2C, 4C and 8C cells in the fat bodies of queens in different stages (QT0 to QT4). The ternary graph shows the actual data points with stages distinguished by a color code. The table lists per stage the average percentages of 2C, 4C, 8C. Number of replicates per group are provided in Supplementary Table 1. The proportions of cells with different DNA content differ between stages (compositional perMANOVA, *p-value* = 0.001), and in particular stages QT0 and QT2 differ from QT4 (post-hoc perMANOVA, both *p-value* = 0.02).

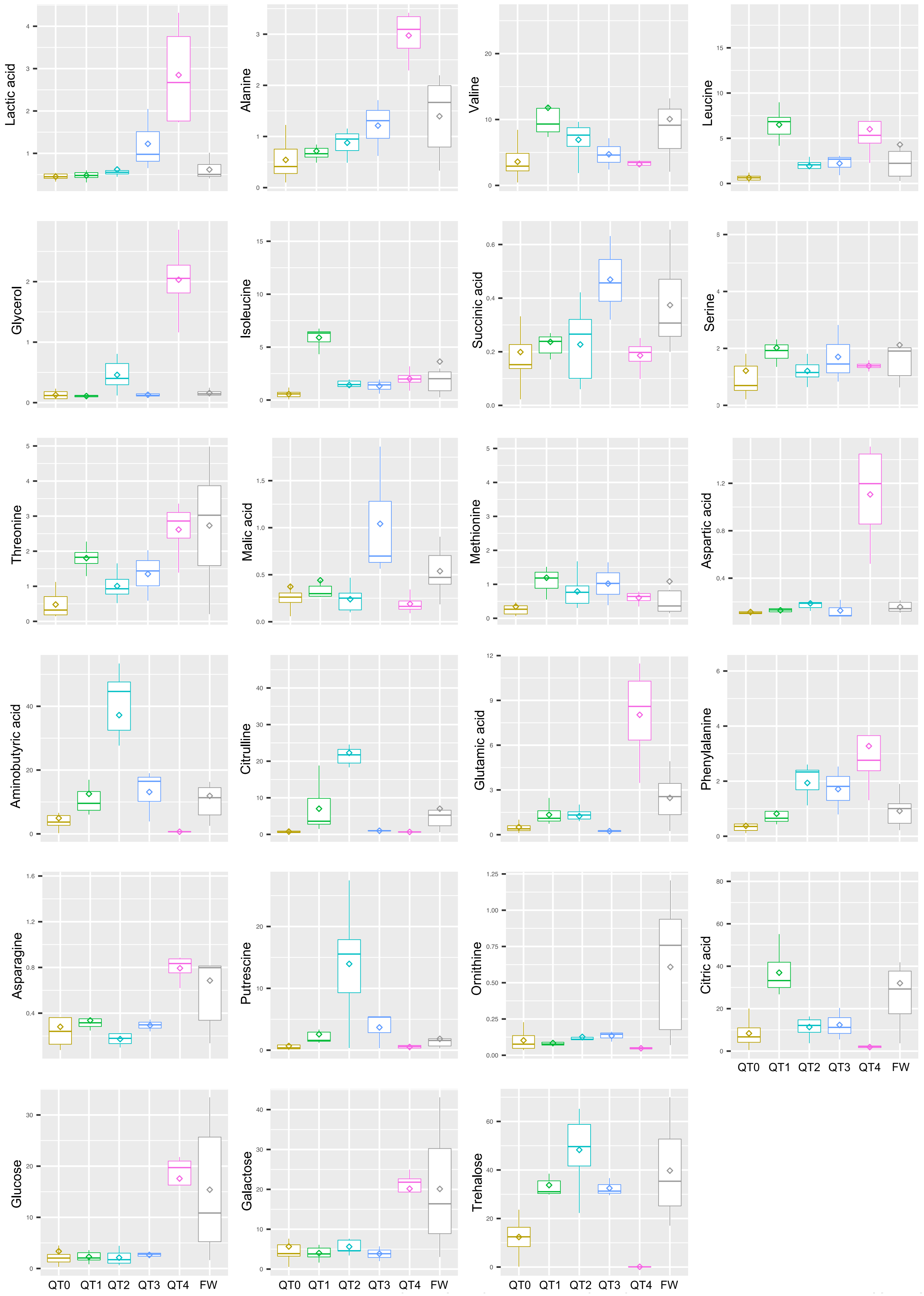

Supplementary Figure 7: Metabolite concentrations (µmol/µl of hemolymph) in five queen maturation stages (QT0-QT4) and female workers (FW). A box consists of upper and lower hinges and a center line corresponding to the 25th percentile, the 75th percentile and the median, respectively. Numbers of replicates per group are provided in supplementary Table 1.

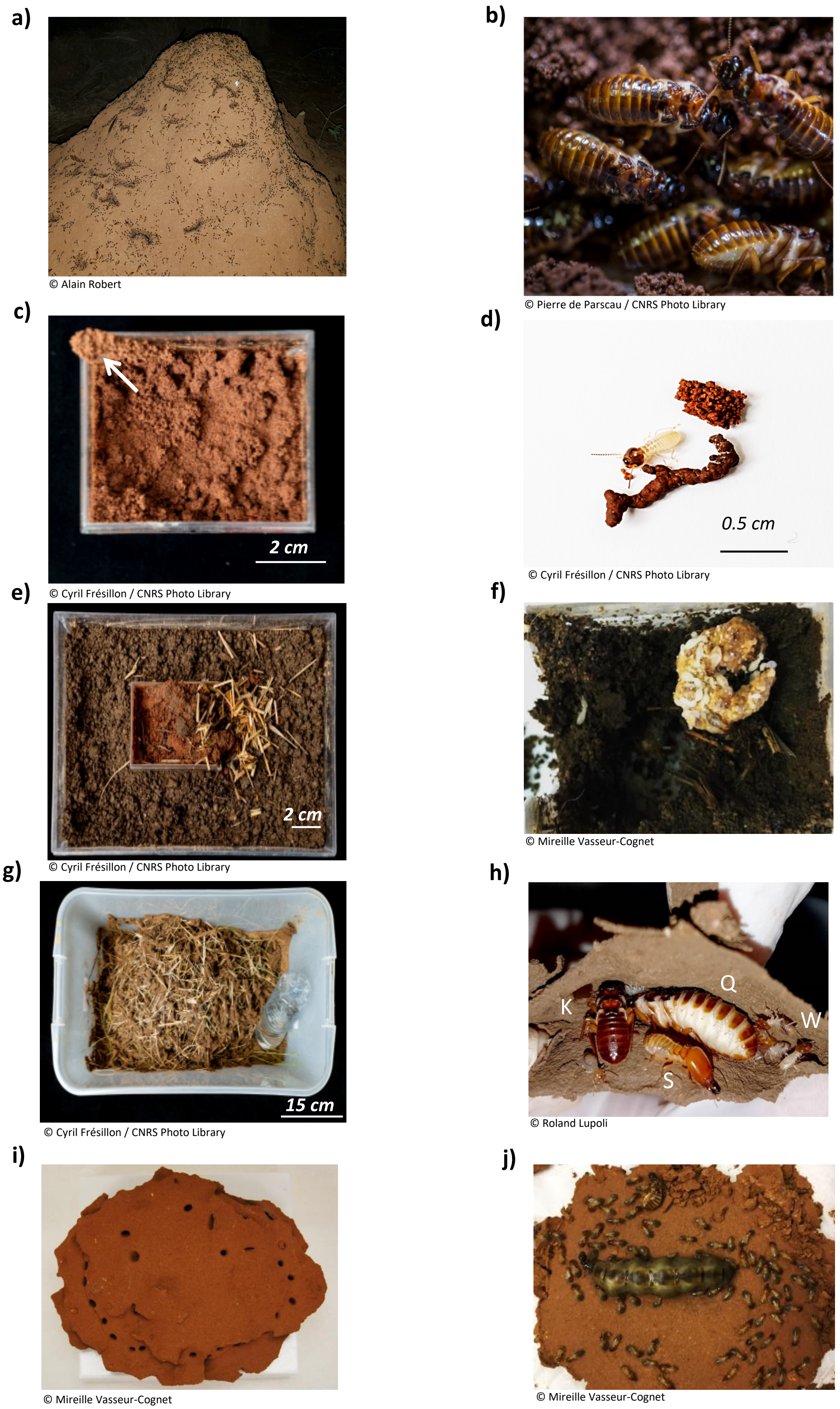

Supplementary Figure 8: Illustrations of colony establishment in the laboratory and mature colonies in the field. Pictures of the different stages of the *Macrotermes natalensis* colonies founded from one male and one female imago each (QT0) each and raised during 31 months (**c-h**) or of the termite colonies collected in the field (**a-b; i-j**). Queens were sampled at 3 months old (QT1), 9 months old (QT2), 31 months old (QT3), and over 20 years old (QT4). Kings (KT4) and FW were sampled from twenty-year-old colonies only. Male and female imagoes collected at the swarm were also sampled (T0). **(a)** Mound with horizontal openings created by workers. **(b)** Imagoes without wings after swarming. **(c)** Three-months old incipient colony with a gallery built by workers and expanding out of the box. **(d)** One worker with two structures, including one to receive the symbiotic fungus, built by workers from the colony in Supplementary Figure 8c. **(e)** Nine-months old incipient colony in a bigger box than the one in Supplementary Figure 8c and fed wild oats only. **(f)** Fungus comb with larvae and workers dug out from the colony shown in Supplementary Figure 8e. **(g)** Thirty-one months old colony in a bigger box than the one from the Supplementary Figure 8e and fed with wild oats and wood. **(h)** The royal couple with sterile castes in the colony from the Supplementary Figure 8g. K, king; Q, queen; W, worker; S, soldier. **(i)** Closed royal chamber with holes from over 20 years old colony. **(j)** Open royal chamber with one physogastric queen (QT4), one king (KT4), and several workers (W) and soldiers (S).

Supplementary Table 1: Summary of the sampling design. This table contains the origin of samples (Field or laboratory, colony ID and year of sampling), number of individuals pooled and number of replicates per group and per experiment.

				FAT BODY			HEMOLYMPH	
	**Castes/stages**	**Year of sampling**	**Colony ID**	**Transcriptomics**	**Lipidomics**	**Ploidy**	**Metabolomics**	**Lipidomics**
FIELD	**FW (adult)**							
	Number of individuals pooled per sample			85			85	85
	Number of samples			4			6	4
		2016	3	X			X	X
			5	X			X	X
			6	X			X	
			7	X			X	X
		2018	Green				X	X
			Blue				X	
			Yellow					
			Red					
	**QT4**							
	Number of individuals pooled per sample			1	1	1	1	1
	Number of samples			4	3	6	4	4
		2016	3	X		X	X	X
			5	X	X	X	X	X
			6	X	X	X	X	X
			7	X			X	X
		2018	Green					
			Blue			X		
			Yellow			X		
			Red		X	X		
	**KT4**							
	Number of individuals pooled per sample			1				
	Number of samples			3				
		2016	3					
			5	X				
			6	X				
			7	X				
		2018	Green					
			Blue					
			Yellow					
			Red					
	**QT0**							
	Number of individuals pooled per sample			10	10	1	10	
	Number of samples			4	4	4	8	
		2016	3	X		X	X	
			5	X	X	X	X	
			6	X	X	X	X	
			7	X		X	X	
		2018	Green		X		X	
			Blue				X	
			Yellow				X	
			Red		X		X	
LABORATOTY	**QT1**							
	Number of individuals pooled per sample			10		1	10	
	Number of samples			4		4	7	
		2016	3	X		X	X	
			5	X		X	X	
			6	X		X	X	
			7	X		X	X	
		2018	Green				X	
			Blue					
			Yellow				X	
			Red				X	
	**QT2**							
	Number of individuals pooled per sample			10	10	1	10	
	Number of samples			3	3	3	7	
		2016	3					
			5				X	
			6		X	X	X	
			7	X			X	
		2018	Green	X	X	X	X	
			Blue	X		X	X	
			Yellow				X	
			Red		X		X	
	**QT3**							
	Number of individuals pooled per sample			1		1	1	
	Number of samples			3		3	3	
		2016	3	X		X	X	
			5					
			6	X		X	X	
			7	X		X	X	
		2018	Green					
			Blue					
			Yellow					
			Red					

Supplementary Table 2: Differential gene expression possibly related to longevity. This table contains genes with significantly different expression between FW and QT4, between FW and KT4. Differences between queens QT4 and KT4 not significant. Numbers of replicates per group are provided in Supplementary Table 1. Annotations include WGCNA module (moduleColor) (Supplementary Fig. 2), gene names and gene acronyms in *Drosophila melanogaster* and *Homo sapiens*. Genes were considered significantly differentially expressed between QT4 and FW, KT4 and FW or KT4 and QT4 if the adjusted *p-value* (adj.P) was less than 0.05.

gene_id	Dmel_Symbol	Hsap_Symbol	Name	moduleColor	FW	QT4	KT4	FWvQT4.adj.P	FWvKT4.adj.P	QT4vKT4.adj.P
**Metabolic signaling pathways**										
Mnat_00258	Ilp9	NA	Insulin-like peptide 9	yellow	-8.024	1.429	0.954	4.14E-108	7.35E-92	
Mnat_00489	Myc	NA	Myc Proto-Oncogene	pink	-1.606	-0.031	0.177	0.00274	0.000464	
Mnat_02791	raptor	RPTOR	Regulatory associated protein of TOR	pink	0.555	-0.889	-0.39	3.26E-05	0.00616	
Mnat_04219	Max	MAX	MYC Associated Factor X	lightcyan	-0.544	0.451	0.07	7.28E-08	0.00547	
Mnat_07221	InR2	NA	Insulin receptor 2	lightgreen	0.3	-0.976	-1.098	8.04E-08	1.95E-05	
Mnat_12310	rictor	NA	RPTOR Independent Companion Of MTOR Complex 2	lightgreen	-0.471	0.069	0.107	0.00518	0.0114	
Mnat_12572	Tor	MTOR	Target of rapamycin	lightgreen	0.722	-0.486	-0.348	2.42E-06	0.000134	
Mnat_13906	eIF6	EIF6	Eukaryotic Translation Initiation Factor 6	lightcyan	-0.298	0.438	0.412	5.22E-05	0.00341	
Mnat_14791	Akt1	AKT3	Protein kinase B	lightgreen	0.415	-1.094	-0.663	2.64E-06	0.00218	
Mnat_15237	S6k	RPS6KB1	S6 kinase	lightgreen	1.105	-1.28	-0.616	6.56E-10	1.47E-05	
**Lipid metabolism**										
Mnat_15516	ELOVL6	Baldspot	Elongation of very long chain fatty acids protein 6	blue	1.307	-0.103	0	3.42E-14	1.16E-08	
Mnat_15428	mdy	NA	Midway/Diacylglycerol O-acyltransferase	lightyellow	0.274	-0.625	-1.281	0.00276	1.36E-07	
**Glycogenesis and trehalose metabolism**										
Mnat_14651	Pgm2a	PGM2	Phosphoglucomutase-2	lightgreen	0.765	-0.279	-0.402	3.65E-05	3.79E-06	
Mnat_11523	GlyS	GYS1	Glycogen synthase	blue	0.861	0.077	-0.353	0.000192	1.55E-07	
**Mitochondria**										
OXPHOS system and assembly										
Mnat_01464	ND-B22	NDUFB9	NADH:Ubiquinone Oxidoreductase Subunit B9	lightcyan	-0.231	0.503	0.547	0.0133	0.0197	
Mnat_03366	CG43346	NDUFAF2	NADH:Ubiquinone Oxidoreductase Complex Assembly Factor 2	lightcyan	-0.731	0.6	0.72	4.44E-05	2.45E-05	
Mnat_04216	CIA30	NDUFAF1	NADH:Ubiquinone Oxidoreductase Complex Assembly Factor 1	lightcyan	-0.009	0.622	0.65	0.0134	0.0218	
Mnat_07542	l(3)87Df	COX20	Cytochrome C Oxidase Assembly Factor†COX20	lightcyan	-0.735	0.887	0.562	3.91E-05	0.00368	
Mnat_12704	CG9065	COX17	Cytochrome C Oxidase Copper Chaperone†COX17	lightcyan	-0.525	0.341	0.51	0.00724	0.00262	
Mitochondrial ribosome										
Mnat_01493	mRpL4	MRPL4	Mitochondrial Ribosomal Protein L4	blue	0.585	0.085	-0.248	0.0279	0.00138	
Mnat_03258	mRpL22	MRPL22	Mitochondrial Ribosomal Protein L22	lightcyan	-0.514	0.415	0.282	0.0108	0.0376	
Mnat_03837	mRpL41	MRPL41	Mitochondrial Ribosomal Protein L41	lightcyan	-0.627	0.809	0.46	1.09E-10	7.62E-05	
Mnat_06399	mRpS14	MRPS14	Mitochondrial Ribosomal Protein S14	lightcyan	-0.508	0.197	0.615	0.00274	1.32E-05	
Mnat_09821	mRpL24	MRPL24	Mitochondrial Ribosomal Protein L24	lightcyan	-0.6	0.379	0.489	0.00041	0.000726	
Mnat_11441	mRpS28	MRPS28	Mitochondrial Ribosomal Protein L28	lightcyan	-0.595	0.215	0.622	0.00387	2.61E-05	
Mnat_12386	mRpL11	MRPL11	Mitochondrial Ribosomal Protein L11	lightcyan	-0.364	0.46	0.422	0.0214	0.0334	
Mnat_13625	mRpL54	MRPL54	Mitochondrial Ribosomal Protein L54	lightcyan	-0.626	0.171	0.101	0.0154	0.0384	
Mnat_13691	mRpS2	MRPS2	Mitochondrial Ribosomal Protein S2	lightcyan	-0.495	0.379	0.287	0.0048	0.0243	
Mnat_14494	CG14817	MRPL57	Mitochondrial Ribosomal Protein L57	lightcyan	-0.764	0.034	0.574	0.036	0.00063	
Mnat_15943	mRpS6	MRPS6	Mitochondrial Ribosomal Protein S6	lightcyan	-0.91	0.469	0.701	7.47E-08	8.14E-10	
Mnat_17881	mRpL34	MRPL34	Mitochondrial Ribosomal Protein L34	lightcyan	-0.717	0.301	0.753	0.000804	5.37E-06	
Mitochondrial fission										
Mnat_06397	Tango11	MFF	Mitochondrial Fission Factor	lightcyan	-0.177	0.325	0.354	0.0205	0.0336	
Mnat_12510	Fis1	FIS1	Fission, Mitochondrial 1	lightcyan	-0.304	0.667	0.346	0.000253	0.0298	
Mitochondrial membrane transport protein										
Mnat_09251	CG14270	TIMM29	Translocase Of Inner Mitochondrial Membrane 29	lightcyan	-0.336	0.486	0.301	0.00367	0.0345	
Mnat_09835	Tim17b	TIMM17B	Translocase Of Inner Mitochondrial Membrane 17B	lightcyan	-0.453	0.631	0.267	2.10E-06	0.00403	
**Oxidative status**										
Mnat_11461	Cat	CAT	Catalase	blue	1.125	-0.102	-0.303	2.09E-05	8.38E-07	
Mnat_02476	Eip71CD	MSRA	Methionine Sulfoxide Reductase A	lightyellow	-0.501	-8.371	-7.255	1.10E-104	3.86E-111	
Mnat_11229	Gss2	GSS	Glutathione synthase	lightyellow	0.913	-0.316	0.152	1.75E-07	0.015	
Mnat_09789	GstD1	NA	Glutathione S transferase D1	blue	1.803	-0.453	-0.556	9.81E-12	2.24E-11	
Mnat_16107	Prx3	PRDX3	Peroxiredoxin 3	blue	1.147	-0.441	-0.455	1.07E-13	1.28E-09	
Mnat_13140	NA	GPX3	Glutathione peroxidase 3	lightcyan	-0.82	0.941	0.68	1.41E-06	7.17E-05	
Mnat_02325	GstS1	HPGDS	Glutathione S transferase S1	blue	0.948	-0.153	-0.046	5.46E-08	0.000154	
Mnat_04257	Prx6005	PRDX6	Peroxiredoxin 6	lightcyan	-0.22	0.361	0.512	0.00113	0.00128	
**DNA damage response, genome stability and telomeres**										
Mnat_06447	Ku80	XRCC5	X-Ray Repair Cross Complementing 5	lightcyan	-0.834	0.328	0.467	3.41E-05	6.10E-05	
Mnat_10935	Xpd	ERCC2	ERCC Excision Repair 2, TFIIH Core Complex Helicase Subunit	lightcyan	-0.531	0.344	0.37	0.00253	0.00596	
Mnat_01130	Ercc1	ERCC1	ERCC Excision Repair 1, Endonuclease Non-Catalytic Subunit	lightcyan	-0.904	0.197	0.453	0.00304	2.36E-05	
Mnat_16250	NA	XRCC6	X-Ray Repair Cross Complementing 6	lightcyan	-0.758	0.278	0.623	0.0082	0.000442	
Mnat_12809	Mlh1	MLH1	MutL Homolog 1	lightcyan	-0.694	-0.019	0.081	0.0246	0.0137	
Mnat_14005	Pms2	PMS2	PMS1 Homolog 2, Mismatch Repair System Component	lightcyan	-1.307	0.333	-0.012	2.71E-12	5.64E-07	
Mnat_15518	PCNA	PCNA	Proliferating Cell Nuclear Antigen	lightcyan	-0.831	0.478	0.088	8.26E-11	0.000158	
Mnat_15672	spel1	MSH2	MutS Homolog 2	lightcyan	-1.489	0.69	0.535	3.25E-24	4.48E-12	
Mnat_04210	spn-A	RAD51	RAD51†Recombinase	red	-1.791	-0.695	0.503	0.0088	2.17E-06	
Mnat_00740	NA	MSH4	MutS Homolog 4	lightcyan	-4.88	-0.487	1.508	3.78E-13	2.14E-48	
Mnat_08389	NA	PMS1	PMS1†Homolog 1, Mismatch Repair System Component	yellow	-0.909	0.722	0.208	7.44E-07	0.0049	
Mnat_12125	RfC3	RFC5	Replication Factor C Subunit 5	lightcyan	-0.651	0.327	0.425	2.59E-05	1.57E-05	
Mnat_00056	RpA-70	RPA1	Replication Protein A1	lightcyan	-0.722	-0.003	0.495	0.000605	7.34E-11	
Mnat_03371	CCT7	CCT7	Chaperonin Containing TCP1 Subunit 7	lightcyan	-0.299	0.192	0.542	0.0191	0.000243	
Mnat_12961	CCT6	CCT6A	Chaperonin Containing TCP1 Subunit 6A	lightcyan	-0.411	0.321	0.675	2.25E-05	1.64E-07	
Mnat_11380	Rhau	DHX36	DEAH-Box Helicase 36	lightcyan	-0.251	0.287	0.592	0.00012	6.97E-07	
Mnat_03394	Sirt6	SIRT6	Sirtuin 6	lightcyan	-0.847	-0.19	0.158	0.033	0.000507	

Supplementary Table 3: Differential gene expression possibly related to longevity and/or to fecundity. This table contains genes where effects are or might be to some extent shared between kings and queens, but less certainly so than for supplementary Table 2. This table contains a subset of genes with significantly different expression between FW and QT4 (not present in Table 2). Among these genes, some genes are significantly different between QT4 and KT4 and between FW and KT4 and signs of effects in kings and queens shared. Within this subset, other genes are not significantly different between QT4 and KT4 but not also between KT4 and FW. Numbers of replicates per group are provided in Supplementary Table 1. Annotations include WGCNA module (moduleColor) (Supplementary Fig. 2), gene names and gene acronyms in *Drosophila melanogaster* and *Homo sapiens*. Genes were considered significantly differentially expressed between QT4 and FW or KT4 and FW if the adjusted *p-value* (adj.P) was less than 0.05.

gene_id	Dmel_Symbol	Hsap_Symbol	Name	moduleColor	FW	QT4	KT4	FWvQT4.adj.P	FWvKT4.adj.P	QT4vKT4.adj.P
**Metabolic signaling pathways**										
Mnat_01205	Pi3K21B	PIK3R3	Phosphatidylinositol 3-kinase	lightgreen	-0.612	0.188	-0.062	0.00675		
Mnat_06530	crc	ATF4	Cryptocephal	lightcyan	-0.977	0.666	-0.261	2.13E-23	0.00376	7.49E-05
Mnat_07472	Pi3K92E	PIK3CD	Phosphatidylinositol 3-kinase	pink	0.388	-1.237	-0.251	0.00122		
Mnat_09744	Pi3K59F	PIK3C3	Phosphatidylinositol 3-kinase	lightcyan	-0.97	0.823	-0.347	1.38E-18	0.0052	9.07E-10
**Lipid metabolism**										
Mnat_00511	vg	NA	Vitellogenin	yellow	-6.395	1.376	-4.428	2.03E-75	0.000386	4.60E-45
**Carbohydrate metabolism**										
Glycolysis										
Mnat_07401	Hex-A	HK2	Hexokinase-A	blue	1.013	0.053	-0.94	1.71E-07	8.00E-28	1.37E-08
Glycogenesis and trehalose metabolism										
Mnat_15718	UGP	UGP2	UTP--glucose-1-phosphate uridylyltransferase	blue	0.74	-0.195	0.241	0.000479		
HBP and O-GlcNAcylation										
Mnat_15532	sxc	OGT	O-GlcNAc transferase	yellow	-0.692	0.887	-0.114	2.63E-07	0.02	0.0115
Mnat_03748	nst	PGM3	Phosphoacetylglucosamine mutase	lightcyan	-0.632	0.93	0.08	7.30E-10	0.0235	1.09E-06
**Mitochondria**										
OXPHOS system and assembly										
Mnat_13408	Stoml2	STOML2	Stomatin Like 2	lightcyan	-0.44	0.736	-0.017	3.87E-07	0.0398	0.00234
Mitochondrial ribosome										
Mnat_08713	mRpL42	MRPL42	Mitochondrial Ribosomal Protein L42	lightcyan	-0.524	0.067	0.714	0.0374	2.36E-05	0.0256
Mnat_09579	mRpL27	MRPL27	Mitochondrial Ribosomal Protein L27	lightcyan	-0.677	0.548	-0.132	8.33E-09	0.0266	0.000962
Mitochondrial membrane transport protein										
Mnat_08042	Tom7	TOMM7	Translocase Of Outer Mitochondrial Membrane 7	yellow	-0.954	0.821	-0.209	3.82E-12	0.0256	7.08E-05
**Oxidative status**										
Mnat_03229	GstO3	GSTO1	Glutathione S-Transferase Omega 1	blue	2.353	-3.333	-4.88	1.11E-106	3.56E-139	2.82E-14
Mnat_09436	Pxn	PXDN	peroxidasin	lightgreen	1.661	-4.113	-2.265	1.11E-36	8.17E-31	0.00781
**DNA damage response, genome stability and telomeres**										
Mnat_12187	kin17	KIN	Kin17 DNA And RNA Binding Protein	lightcyan	-0.773	0.843	-0.04	4.72E-16	0.000778	5.01E-05
Mnat_09905	Nap1	NAP1L1	Nucleosome Assembly Protein 1 Like 1	lightcyan	-0.881	-0.076	0.757	0.0071	2.89E-07	2.99E-05
Mnat_11092	NA	TP53BP1	Tumor Protein P53 Binding Protein 1	lightcyan	-1.755	0.114	0.988	2.66E-06	2.62E-12	0.03
Mnat_03549	rad50	RAD50	RAD50†Double Strand Break Repair Protein	lightgreen	-1.505	-0.336	0.308	1.16E-07	6.81E-18	0.038
Mnat_17206	RfC38	RFC3	Replication Factor C Subunit 3	lightcyan	-1.137	0.405	-0.262	2.65E-10	0.00192	0.00887
Mnat_10872	RPA2	RPA2	Replication Protein A2	lightcyan	-1.113	-0.327	0.481	0.0266	7.72E-06	0.00124
Mnat_01158	CCT4	CCT4	Chaperonin Containing TCP1 Subunit 4	lightcyan	-0.678	0.535	0.157	1.10E-07	0.00274	0.013

Supplementary Table 4: Differential gene expression possibly related to queen fecundity. This table contains two groups: (1) Genes with significantly different expression among the three groups (FW, QT4, KT4) and whose signs of effects in kings and queens opposite. (2) gene significantly different between QT4 versus KT4 and between QT4 versus FW and not between FW and KT4 (genes not in Supplementary Tables 2 and 3). Numbers of replicates per group are provided in Supplementary Table 1. Annotations include WGCNA module (moduleColor) (Supplementary Fig. 2), gene names and gene acronyms in *Drosophila melanogaster* and *Homo sapiens* . Genes were considered significantly differentially expressed between QT4 and FW or KT4 and FW if the adjusted *p-value* (adj.P) was less than 0.05.

gene_id	Dmel_Symbol	Hsap_Symbol	Name	moduleColor	FW	QT4	KT4	FWvQT4.adj.P	FWvKT4.adj.P	QT4vKT4.adj.P
**Metabolic signaling pathways**										
Mnat_00349	NA	NA	Insulin receptor 3	lightgreen	-0.869	0.438	-1.753	0.000244	0.0242	1.89E-13
Mnat_06881	Pdk1	PDPK1	Phosphoinositide-dependent kinase-1	lightgreen	-0.342	0.689	-1.549	8.69E-05	7.87E-05	2.75E-23
**Lipid metabolism**										
Lipogenesis										
Mnat_02170	ACC	ACACA	Acetyl-CoA carboxylase	yellow	-1.223	0.565	-1.245	4.02E-05		1.50E-08
Mnat_12194	FASN1	NA	Fatty acid synthase	yellow	-1.438	1.505	-0.984	1.66E-22		3.85E-15
FA activation, elongation, esterification, and trafficking										
Mnat_09846	bgm	ACSBG2	Bubblegum/Long-chain-fatty-acid--CoA ligase	yellow	-0.452	1.443	-1.202	1.72E-14	0.0148	3.83E-38
Mnat_10187	CG3961	ACSL1	Acyl CoA long chain ligase	lightyellow	0.543	-3.257	1.074	1.50E-27	0.0439	3.19E-26
Mnat_15087	Gpat4	GPAT4	Glycerol-3-phosphate acyltransferase 4	lightgreen	-0.915	0.722	-1.315	1.24E-08		6.89E-12
Mnat_04243	Lpin	LPIN1	Lipin/Phosphatidate phosphatase	yellow	-0.405	1	-0.861	0.000653		1.87E-14
Mnat_01743	Desat1	SCD1	Desaturase 1	yellow	-0.891	1.395	-0.853	2.00E-28		2.59E-33
Mnat_04621	NA	HDLBP	Diacylglycerol-carrying lipoprotein	yellow	-0.205	0.789	-1.174	6.24E-12	8.98E-06	4.16E-49
Mnat_08606	Gk1	GK	Glycerol kinase	yellow	-0.638	1.396	-0.335	3.65E-13		5.12E-21
Mnat_11814	AcCoAS	ACSS2	Acyl-CoA Synthetase Short Chain Family Member	yellow	-0.972	1.695	-1.36	4.76E-17		2.04E-29
**Carbohydrate metabolism**										
Glycolysis										
Mnat_01413	CG6650	ADPGK	ADP-dependant glucokinase	lightcyan	0.132	0.64	-0.628	0.0298	0.00779	9.80E-12
Mnat_01955	Pfk	PFKM	Phosphofructokinase	lightcyan	-0.131	0.845	0.059	0.000465		0.0215
Mnat_09840	PyK	PKM	Pyruvate kinase	lightcyan	-0.264	0.955	0.007	3.73E-07		0.000139
Mnat_04297	PDK3	Pdk	Pyruvate dehydrogenase kinase 3	lightyellow	-0.677	-4.345	-0.644	3.29E-16		1.62E-15
Trehalose metabolism										
Mnat_01204	Tps1	NA	Trehalose-6-phosphate synthase	lightgreen	0.154	-1.389	-0.333	5.26E-09		0.000722
HBP and O-GlcNAcylation										
Mnat_03461	Oscillin	GNPDA2	Glucosamine-6-phosphate deaminase	yellow	-0.703	0.832	-0.467	3.60E-27		1.72E-08
Mnat_14855	Gnpnat	GNPNAT1	Glucosamine-phosphate N-acetyltransferase	yellow	-0.958	0.97	-1.128	1.03E-24		2.32E-19
Mnat_06338	CG31637	CHST5	N-acetylglucosamine 6-O-sufotransferase	yellow	-1.989	0.469	-1.597	7.50E-16		1.81E-08
Pentose phosphate pathway										
Mnat_01614	CG8036	TKT	Transketolase	yellow	-0.202	0.778	-1.088	4.32E-06	0.000185	3.38E-23

Supplementary Table 5: Metabolite concentrations (µmol/µl of hemolymph) in five adult queen maturation stages (QT0-QT4) and female workers (FW). Average concentrations per metabolite and caste or stage are given. In addition, adjusted tail probabilities (Benjamini-Hochberg adjusted for multiple comparisons) of Welch tests comparing each metabolite between adjacent queen maturation stages, between female workers and virgin queens (QT0), female workers (FW) and highly fertile (QT4) queens. Metabolites are ordered according their average concentration in QT4. Number of replicates per group is provided in supplementary Table 1.

	Average concentrations						Adjusted p-values					
**Metabolites**	**QT0**	**QT1**	**QT2**	**QT3**	**QT4**	**FW**	**QT1 vs QT0**	**QT2 vs QT1**	**QT3 vs QT2**	**QT4 vs QT3**	**QT0 vs FW**	**QT4 vs FW**
Lactic acid	0.45	0.48	0.62	1.23	2.85	0.62						1.10E-02
Alanine	0.54	0.72	0.88	1.21	2.97	1.4						4.20E-02
Valine	3.59	11.79	6.93	4.73	3.22	10.07	9.00E-03					
Leucine	0.62	6.5	1.93	2.22	6	4.3	0.00E+00	3.00E-03				
Glycerol	0.13	0.11	0.46	0.13	2.03	0.16		5.00E-03	4.70E-02	2.00E-03		0.00E+00
Isoleucine	0.56	5.9	1.42	1.32	2.01	3.64	1.00E-03	3.00E-03				
Succinic acid	0.2	0.24	0.23	0.47	0.19	0.37				4.10E-02		
Serine	1.22	2.02	1.21	1.7	1.39	2.12		2.90E-02				
Threonine	0.48	1.8	1.01	1.35	2.62	2.73	4.00E-03	1.60E-02				
Malic acid	0.37	0.44	0.24	1.04	0.19	0.54				4.10E-02		4.20E-02
Methionine	0.34	1.19	0.79	1.02	0.6	1.09	9.00E-03					
Aspartic acid	0.12	0.13	0.19	0.13	1.11	0.16				2.20E-02		8.00E-03
Aminobutyric acid	4.94	12.55	37.23	13.14	0.68	11.91				4.60E-02		0.00E+00
Citrulline	0.77	7.03	22.28	1.01	0.68	7.02	4.00E-03		5.00E-03	3.20E-02		4.10E-02
Glutamic acid	0.51	1.34	1.25	0.24	8.03	2.47	1.30E-02		1.50E-02	2.00E-03		4.20E-02
Phenylalanine	0.38	0.82	1.94	1.71	3.27	0.92	2.80E-02					4.10E-02
Asparagine	0.28	0.34	0.17	0.29	0.79	0.69		5.00E-03		7.00E-03		
Putrescine	0.68	2.58	13.96	3.69	0.53	1.84	2.00E-02					
Ornithine	0.1	0.08	0.13	0.13	0.05	0.61		2.90E-02		3.90E-02		1.40E-02
Citric acid	8.36	36.97	11.33	12.34	1.91	31.99	9.00E-03	3.00E-03		4.00E-02		4.00E-03
Glucose	3.37	2.28	2.13	2.66	17.58	15.4				4.00E-03		
Galactose	5.67	4.02	5.62	3.85	20.17	20.11				3.90E-02		
Trehalose	12.38	33.78	48.29	32.51	0.11	39.75				4.00E-03		0.00E+00

Supplementary Table 6: Lipid concentrations in hemolymph of female workers (FW) and long-lived queens (QT4) shown in Figure 7. Data are expressed in ion abundance normalized by an internal standard. Numbers of replicates per group are provided in Supplementary Table 1. For each lipid, p-adjust (adj.P) indicate significantly different values according to Welch tests. Benjamini-Hochberg's corrections for multiple comparisons were applied. Lipid concentrations were considered significantly different if the adjusted *p-value* (adj.P) was less than 0.05.

Lipid	FW		QT4		adj.P
	Mean	SD	Mean	SD	
DG 34:1|DG 16:0_18:1	5.E+02	2.E+02	7.E+04	4.E+04	0.00E+00
DG 34:2|DG 16:0_18:2	4.E+02	2.E+02	2.E+04	8.E+03	0.00E+00
DG 36:1|DG 18:0_18:1	6.E+02	2.E+02	4.E+04	2.E+04	0.00E+00
DG 36:2|DG 18:1_18:1	1.E+03	5.E+02	8.E+04	4.E+04	0.00E+00
DG 36:3|DG 18:1_18:2	1.E+03	6.E+02	7.E+04	3.E+04	0.00E+00
DG 36:4|DG 18:1_18:3	3.E+02	2.E+02	1.E+04	7.E+03	0.001
LPC 16:0	6.E+02	5.E+02	1.E+02	8.E+01	
LPC 18:1/0:0	5.E+03	4.E+03	7.E+02	3.E+02	
LPC 18:2	5.E+02	4.E+02	1.E+01	8.E+00	2.90E-02
LPC 20:4/0:0	3.E+02	3.E+02	1.E+01	9.E+00	
PC 32:0|PC 16:0_16:0	6.E+02	3.E+02	2.E+03	9.E+02	
PC 34:1|PC 16:0_18:1	3.E+04	2.E+04	2.E+05	7.E+04	1.30E-02
PC 34:2|PC 16:0_18:2	1.E+04	6.E+03	9.E+03	7.E+02	
PC 36:1|PC 18:0_18:1	7.E+03	2.E+03	3.E+04	1.E+03	1.20E-02
PC 36:2|PC 18:1_18:1	6.E+04	3.E+04	2.E+05	3.E+04	3.20E-02
PC 36:3|PC 18:1_18:2	3.E+04	1.E+04	1.E+04	2.E+03	
PC 36:4|PC 18:1_18:3	4.E+03	4.E+03	1.E+04	4.E+03	
PC 36:4|PC 18:2_18:2	3.E+03	1.E+03	1.E+03	2.E+02	3.60E-02
PC 36:5|PC 18:2_18:3	6.E+02	4.E+02	6.E+02	2.E+02	
PC 38:4|PC 18:0_20:4	3.E+02	2.E+02	3.E+03	4.E+02	1.80E-02
PC 38:5|PC 18:1_20:4	8.E+03	5.E+03	9.E+03	4.E+02	
PC 38:6|PC 18:2_20:4	3.E+03	1.E+03	1.E+03	4.E+02	
PE 34:1|PE 16:0_18:1	8.E+02	4.E+02	1.E+03	4.E+02	
PE 36:1|PE 18:0_18:1	8.E+02	4.E+02	2.E+03	1.E+03	
PE 36:2	5.E+02	2.E+02	6.E+02	2.E+02	
PE 36:2|PE 18:1_18:1	3.E+03	1.E+03	7.E+03	4.E+03	
PE 36:3|PE 18:1_18:2	1.E+03	8.E+02	6.E+02	3.E+02	
SM 36:1;2O|SM 14:1;2O/22:0	1.E+02	6.E+01	8.E+02	4.E+02	1.50E-02
SM 36:2;2O	1.E+02	4.E+01	1.E+03	4.E+02	1.00E-03
TG 44:1|TG 12:0_16:0_16:1	8.E+01	2.E+01	8.E+01	2.E+01	
TG 46:1|TG 12:0_16:0_18:1	3.E+02	8.E+01	2.E+02	7.E+01	
TG 46:1|TG 12:0_16:0_18:1	6.E+01	2.E+01	5.E+01	2.E+01	
TG 46:2|TG 12:0_16:0_18:2	4.E+01	2.E+01	4.E+01	3.E+01	
TG 46:2|TG 12:0_16:0_18:2	3.E+01	1.E+01	2.E+01	1.E+01	
TG 47:1|TG 13:0_16:0_18:1	5.E+02	2.E+02	3.E+02	3.E+01	
TG 48:0|TG 14:0_16:0_18:0	1.E+03	8.E+02	6.E+02	1.E+02	
TG 48:1|TG 14:0_16:0_18:1	1.E+03	7.E+02	6.E+02	9.E+01	
TG 48:1|TG 14:0_16:0_18:1	3.E+02	2.E+02	2.E+02	2.E+01	
TG 48:2|TG 14:0_16:0_18:2	1.E-02	5.E-03	6.E-03	1.E-03	
TG 48:2|TG 14:0_16:1_18:1	4.E+02	2.E+02	2.E+02	1.E+01	
TG 48:3|TG 12:0_18:1_18:2	6.E+01	3.E+01	2.E+01	5.E+00	1.60E-02
TG 48:3|TG 12:0_18:1_18:2	2.E+01	2.E+01	1.E+01	2.E+00	
TG 49:1|TG 13:0_18:0_18:1	2.E+03	6.E+02	9.E+02	1.E+02	3.60E-02
TG 49:2|TG 15:0_16:0_18:2	4.E+02	2.E+02	2.E+02	3.E+01	2.80E-02
TG 49:3|TG 15:0_16:1_18:2	7.E+01	3.E+01	2.E+01	5.E+00	1.40E-02
TG 50:1|TG 16:0_16:0_18:1	8.E+02	1.E+03	5.E+02	7.E+02	
TG 50:1|TG 16:0_16:0_18:1	1.E+04	7.E+03	5.E+03	2.E+03	
TG 50:2|TG 16:0_16:1_18:1	5.E+03	4.E+03	1.E+03	5.E+02	
TG 50:3|TG 16:0_16:1_18:2	5.E+02	3.E+02	1.E+02	7.E+00	
TG 50:3|TG 16:0_16:1_18:2	2.E+02	1.E+02	5.E+01	1.E+01	
TG 50:4|TG 14:0_16:0_20:4	3.E+01	2.E+01	7.E+00	5.E+00	3.30E-02
TG 50:4|TG 16:0_16:1_18:3	5.E+01	3.E+01	2.E+01	5.E+00	3.60E-02
TG 51:1|TG 16:0_17:0_18:1	4.E+03	2.E+03	9.E+02	3.E+02	2.40E-02
TG 51:2|TG 16:0_17:1_18:1	3.E+03	1.E+03	5.E+02	1.E+02	1.60E-02
TG 51:3|TG 15:0_18:1_18:2	5.E+02	3.E+02	8.E+01	1.E+01	2.40E-02
TG 51:4|TG 16:0_17:1_18:3	8.E+01	3.E+01	1.E+01	6.E+00	1.20E-02
TG 52:1|TG 16:0_18:0_18:1	2.E+04	9.E+03	6.E+03	2.E+03	
TG 52:2|TG 16:0_18:1_18:1	2.E+03	2.E+03	1.E+03	2.E+03	
TG 52:2|TG 16:0_18:1_18:1	3.E+04	3.E+04	1.E+04	6.E+03	
TG 52:3|TG 16:0_18:1_18:2	1.E+04	9.E+03	2.E+03	1.E+03	
TG 52:4|TG 16:1_18:1_18:2	1.E+03	6.E+02	4.E+02	8.E+01	
TG 52:4|TG 16:1_18:1_18:2	4.E+02	3.E+02	2.E+02	3.E+01	
TG 52:5|TG 16:0_18:2_18:3	1.E+02	5.E+01	9.E+01	4.E+01	
TG 52:5|TG 16:0_18:2_18:3	1.E+02	3.E+01	6.E+01	7.E+00	
TG 53:1|TG 17:0_18:0_18:1	1.E+03	6.E+02	2.E+02	3.E+01	1.60E-02
TG 53:2|TG 17:0_18:0_18:2	3.E+03	2.E+03	5.E+02	1.E+02	1.40E-02
TG 53:3|TG 17:0_18:1_18:2	2.E+03	8.E+02	2.E+02	4.E+01	1.60E-02
TG 53:4|TG 17:1_18:1_18:2	4.E+02	2.E+02	6.E+01	3.E+01	1.20E-02
TG 54:1|TG 18:0_18:0_18:1	6.E+03	1.E+03	3.E+03	4.E+02	1.60E-02
TG 54:2|TG 18:0_18:1_18:1	1.E+04	6.E+03	4.E+03	1.E+03	
TG 54:3|TG 18:1_18:1_18:1	2.E+04	2.E+04	6.E+03	4.E+03	
TG 54:4|TG 18:1_18:1_18:2	8.E+03	7.E+03	2.E+03	1.E+03	
TG 54:5|TG 18:1_18:2_18:2	2.E+03	7.E+02	8.E+02	1.E+02	
TG 54:6|TG 18:1_18:2_18:3	4.E+02	1.E+02	3.E+02	7.E+01	
TG 55:2|TG 18:0_19:0_18:2	8.E+02	4.E+02	9.E+01	1.E+01	8.00E-03
TG 55:3|TG 19:0_18:1_18:2	1.E+03	4.E+02	1.E+02	5.E+01	5.00E-03
TG 56:1|TG 18:0_20:0_18:1	6.E+02	2.E+02	3.E+02	1.E+02	
TG 56:2|TG 20:0_18:1_18:1	1.E+03	6.E+02	4.E+02	6.E+01	2.40E-02
TG 56:3|TG 20:0_18:1_18:2	1.E+03	7.E+02	2.E+02	8.E+01	3.80E-02
TG 58:1|TG 18:0_22:0_18:1	3.E+02	8.E+01	2.E+02	8.E+01	
TG 58:2|TG 22:0_18:1_18:1	3.E+02	6.E+01	1.E+02	4.E+01	2.10E-02

Supplementary Table 7: Lipid concentrations in fat body of queen at different stages (QT0, QT2 and QT4) shown in Figure 8. Data are expressed in ion abundance normalized by an internal standard. Numbers of replicates per group are provided in Supplementary Table 1. For each lipid, adj.P indicate significantly different values according to Welch tests. Benjamini-Hochberg's corrections for multiple comparisons were applied. Lipid concentrations were considered significantly different if the adjusted *p-value* (adj.P) was less than 0.05.

Lipid	QT0		QT2		QT4		adj.P		
	Mean	SD	Mean	SD	Mean	SD	QT0 vs QT2	QT2 vs QT4	QT0 vs QT4
DG 34:1|DG 16:0_18:1	2.10E+04	9.20E+03	2.50E+04	2.40E+04	6.10E+04	2.40E+04			
DG 34:2|DG 16:0_18:2	7.70E+03	2.70E+03	9.60E+03	1.20E+04	1.30E+04	7.60E+03			
DG 36:1|DG 18:0_18:1	7.00E+03	2.90E+03	1.30E+04	1.20E+04	3.80E+04	1.90E+04			4.79E-02
DG 36:2|DG 18:1_18:1	2.70E+04	1.30E+04	3.40E+04	3.10E+04	7.80E+04	3.50E+04			
DG 36:3|DG 18:1_18:2	1.10E+04	4.30E+03	2.10E+04	2.20E+04	4.50E+04	2.20E+04			
DG 36:4|DG 18:1_18:3	8.40E+02	3.10E+02	2.30E+03	2.40E+03	7.70E+03	5.00E+03			
LPC 16:0	7.30E+02	3.50E+02	1.00E+04	1.70E+04	8.20E+03	7.90E+03			
LPC 18:1/0:0	1.60E+03	6.80E+02	6.40E+03	7.70E+03	3.80E+04	5.40E+04			
LPC 18:2	1.00E+02	1.60E+01	7.00E+02	1.00E+03	2.00E+03	1.70E+03			
LPC 20:4/0:0	4.80E+01	3.20E+01	1.50E+03	2.40E+03	5.00E+03	5.60E+03			
PC 32:0|PC 16:0_16:0	5.40E+04	1.90E+04	5.10E+03	6.90E+03	1.00E+04	3.60E+03			1.83E-02
PC 34:1|PC 16:0_18:1	7.90E+05	1.50E+05	3.30E+05	3.00E+05	8.00E+05	2.60E+05			
PC 34:2|PC 16:0_18:2	2.10E+05	6.00E+04	2.60E+04	2.10E+04	5.40E+04	2.40E+04			
PC 36:1|PC 18:0_18:1	1.60E+05	2.30E+04	2.60E+04	3.80E+04	2.00E+05	8.10E+04			
PC 36:2|PC 18:1_18:1	6.80E+05	1.40E+05	3.60E+05	3.10E+05	7.30E+05	2.40E+05			
PC 36:3|PC 18:1_18:2	3.40E+05	6.60E+04	2.50E+04	3.30E+04	9.30E+04	3.70E+04			
PC 36:4|PC 18:1_18:3	3.10E+04	7.60E+03	1.20E+04	1.50E+04	3.50E+04	1.20E+04			
PC 36:4|PC 18:2_18:2	1.80E+04	4.70E+03	2.60E+03	2.00E+03	4.00E+03	1.80E+03			4.79E-02
PC 36:5|PC 18:2_18:3	1.70E+04	3.80E+03	1.80E+03	1.60E+03	4.30E+03	2.10E+03			
PC 38:4|PC 18:0_20:4	1.20E+04	1.90E+03	1.50E+03	1.40E+03	1.30E+04	3.90E+03			
PC 38:5|PC 18:1_20:4	1.80E+04	4.90E+03	2.40E+04	1.90E+04	2.10E+04	6.90E+03			
PC 38:6|PC 18:2_20:4	2.50E+04	4.40E+03	2.20E+03	1.80E+03	9.20E+03	3.70E+03			
PE 34:1|PE 16:0_18:1	3.60E+04	7.90E+03	2.40E+04	2.50E+04	3.30E+04	1.40E+04			
PE 36:1|PE 18:0_18:1	5.80E+04	7.20E+03	6.50E+04	6.60E+04	9.40E+04	4.40E+04			
PE 36:2	5.00E+03	1.50E+03	2.20E+04	2.40E+04	6.40E+03	2.40E+03			
PE 36:2|PE 18:1_18:1	1.10E+05	1.50E+04	1.00E+05	1.00E+05	1.90E+05	7.10E+04			
PE 36:3|PE 18:1_18:2	3.60E+04	7.40E+03	1.40E+04	1.30E+04	1.90E+04	8.40E+03			
SM 36:1;2O|SM 14:1;2O/22:0	7.00E+03	1.50E+03	1.60E+04	1.20E+04	5.10E+03	1.80E+03			
SM 36:2;2O	5.70E+03	7.30E+02	3.60E+04	3.50E+04	1.20E+04	2.50E+03			4.79E-02
TG 44:1|TG 12:0_16:0_16:1	3.00E+04	6.70E+03	1.40E+04	1.30E+04	2.80E+03	1.90E+03			
TG 46:1|TG 12:0_16:0_18:1	2.60E+05	6.30E+04	7.20E+04	5.60E+04	3.40E+04	2.50E+04			
TG 46:1|TG 12:0_16:0_18:1	1.40E+04	3.90E+03	2.90E+04	2.50E+04	9.60E+03	7.20E+03			
TG 46:2|TG 12:0_16:0_18:2	8.00E+04	1.90E+04	1.30E+04	1.10E+04	2.30E+03	1.40E+03			
TG 46:2|TG 12:0_16:0_18:2	1.30E+04	4.20E+03	8.10E+03	7.30E+03	1.30E+03	8.90E+02			
TG 47:1|TG 13:0_16:0_18:1	3.60E+04	9.90E+03	5.10E+04	5.00E+04	1.40E+04	8.90E+03			
TG 48:0|TG 14:0_16:0_18:0	5.80E+03	1.80E+03	1.70E+05	1.70E+05	1.20E+04	6.40E+03			
TG 48:1|TG 14:0_16:0_18:1	4.10E+05	1.00E+05	3.60E+05	2.80E+05	1.00E+05	7.60E+04			
TG 48:1|TG 14:0_16:0_18:1	1.00E+04	2.80E+03	9.60E+04	9.40E+04	1.30E+04	8.50E+03			
TG 48:2|TG 14:0_16:0_18:2	7.40E+03	8.10E+02	1.30E+04	1.10E+04	5.10E+03	4.50E+03			
TG 48:2|TG 14:0_16:1_18:1	4.20E+05	1.30E+05	8.70E+04	6.60E+04	3.10E+04	1.90E+04			
TG 48:3|TG 12:0_18:1_18:2	9.00E+04	2.80E+04	8.20E+03	6.90E+03	1.60E+03	8.80E+02			1.01E-02
TG 48:3|TG 12:0_18:1_18:2	1.40E+04	5.20E+03	3.80E+03	3.00E+03	8.90E+02	5.40E+02			
TG 49:1|TG 13:0_18:0_18:1	8.00E+04	2.10E+04	1.00E+05	8.10E+04	2.70E+04	1.80E+04			
TG 49:2|TG 15:0_16:0_18:2	1.30E+05	3.30E+04	3.80E+04	2.80E+04	1.10E+04	6.40E+03			
TG 49:3|TG 15:0_16:1_18:2	2.40E+04	7.80E+03	5.30E+03	4.90E+03	8.00E+02	4.00E+02			1.83E-02
TG 50:1|TG 16:0_16:0_18:1	3.30E+04	3.40E+04	5.90E+04	7.50E+04	4.60E+03	3.10E+03			
TG 50:1|TG 16:0_16:0_18:1	4.90E+05	1.40E+05	1.80E+06	1.40E+06	4.40E+05	2.80E+05			
TG 50:2|TG 16:0_16:1_18:1	9.50E+05	2.70E+05	7.80E+05	5.90E+05	1.70E+05	1.10E+05			
TG 50:3|TG 16:0_16:1_18:2	5.10E+05	1.50E+05	7.10E+04	5.30E+04	9.30E+03	4.60E+03			8.59E-03
TG 50:3|TG 16:0_16:1_18:2	1.90E+04	7.10E+03	3.50E+04	3.00E+04	3.60E+03	1.90E+03			
TG 50:4|TG 14:0_16:0_20:4	1.10E+04	4.40E+03	3.80E+03	3.30E+03	4.50E+02	2.00E+02			9.08E-03
TG 50:4|TG 16:0_16:1_18:3	6.60E+04	2.60E+04	6.80E+03	5.90E+03	9.10E+02	4.70E+02			6.79E-03
TG 51:1|TG 16:0_17:0_18:1	3.30E+04	9.50E+03	9.60E+04	7.40E+04	2.70E+04	1.70E+04			
TG 51:2|TG 16:0_17:1_18:1	1.90E+05	5.20E+04	1.10E+05	8.50E+04	2.60E+04	2.20E+04			
TG 51:3|TG 15:0_18:1_18:2	1.90E+05	5.90E+04	2.60E+04	1.90E+04	4.60E+03	2.30E+03			9.08E-03
TG 51:4|TG 16:0_17:1_18:3	3.30E+04	1.30E+04	3.50E+03	2.80E+03	5.50E+02	2.10E+02			5.83E-03
TG 52:1|TG 16:0_18:0_18:1	2.90E+05	9.00E+04	2.10E+06	1.60E+06	4.80E+05	2.60E+05			
TG 52:2|TG 16:0_18:1_18:1	8.70E+04	2.20E+04	1.70E+05	2.20E+05	1.30E+04	1.10E+04			
TG 52:2|TG 16:0_18:1_18:1	9.30E+05	7.10E+05	3.40E+06	2.70E+06	9.10E+05	5.00E+05			
TG 52:3|TG 16:0_18:1_18:2	1.10E+06	3.40E+05	9.40E+05	7.00E+05	9.00E+04	1.00E+05			
TG 52:4|TG 16:1_18:1_18:2	5.60E+05	1.60E+05	9.20E+04	7.40E+04	1.20E+04	6.20E+03			9.08E-03
TG 52:4|TG 16:1_18:1_18:2	2.20E+04	7.70E+03	4.60E+04	4.50E+04	4.80E+03	2.50E+03			
TG 52:5|TG 16:0_18:2_18:3	9.90E+04	4.40E+04	1.80E+04	2.20E+04	1.30E+03	6.50E+02			5.83E-03
TG 52:5|TG 16:0_18:2_18:3	1.30E+04	5.20E+03	1.10E+04	1.40E+04	7.10E+02	2.90E+02			8.59E-03
TG 53:1|TG 17:0_18:0_18:1	1.70E+04	6.50E+03	1.10E+05	8.70E+04	2.90E+04	1.60E+04			
TG 53:2|TG 17:0_18:0_18:2	8.10E+04	2.40E+04	1.10E+05	8.90E+04	4.00E+04	2.40E+04			
TG 53:3|TG 17:0_18:1_18:2	1.20E+05	3.60E+04	5.90E+04	4.80E+04	1.50E+04	7.20E+03			
TG 53:4|TG 17:1_18:1_18:2	7.50E+04	2.50E+04	1.30E+04	9.30E+03	3.00E+03	1.20E+03			8.59E-03
TG 54:1|TG 18:0_18:0_18:1	1.30E+05	3.70E+04	1.20E+06	9.40E+05	4.30E+05	2.20E+05			
TG 54:2|TG 18:0_18:1_18:1	9.70E+05	2.70E+05	2.20E+06	1.70E+06	8.10E+05	4.70E+05			
TG 54:3|TG 18:1_18:1_18:1	8.60E+05	2.90E+05	1.90E+06	1.40E+06	5.30E+05	2.70E+05			
TG 54:4|TG 18:1_18:1_18:2	6.50E+05	2.10E+05	5.40E+05	4.00E+05	1.00E+05	3.90E+04			2.48E-02
TG 54:5|TG 18:1_18:2_18:2	3.60E+05	1.20E+05	1.00E+05	1.00E+05	1.50E+04	6.70E+03			8.59E-03
TG 54:6|TG 18:1_18:2_18:3	6.30E+04	4.00E+04	3.40E+04	4.70E+04	2.30E+03	1.00E+03			
TG 55:2|TG 18:0_19:0_18:2	2.10E+04	7.30E+03	4.70E+04	3.80E+04	2.00E+04	1.20E+04			
TG 55:3|TG 19:0_18:1_18:2	1.60E+04	5.30E+03	2.40E+04	1.80E+04	8.40E+03	4.90E+03			
TG 56:1|TG 18:0_20:0_18:1	1.20E+04	4.30E+03	1.00E+05	7.80E+04	1.20E+05	6.20E+04			
TG 56:2|TG 20:0_18:1_18:1	1.60E+05	5.20E+04	1.70E+05	1.40E+05	2.10E+05	1.30E+05			
TG 56:3|TG 20:0_18:1_18:2	1.90E+04	6.40E+03	3.80E+04	3.30E+04	1.50E+04	6.40E+03			
TG 58:1|TG 18:0_22:0_18:1	2.20E+03	7.00E+02	2.40E+04	2.10E+04	1.30E+04	6.30E+03			
TG 58:2|TG 22:0_18:1_18:1	1.20E+04	3.90E+03	2.60E+04	2.40E+04	1.10E+04	6.90E+03			

Supplementary Table 8: GPS localizations of *Macrotermes natalensis* colonies sampled in the field in Pretoria (South Africa). The sex of imagoes used for the establishment of laboratory colonies is indicated

Colony ID	GPS coordinates	Sex of imagoes used for colony foundation	Collecting dates
2	25° 73’47.0"S 028°23’98.7"E	Males	October 2016 and 2018
3	25°44'05.0"S 028°14'16.83"E	Females	October 2016
5	25°44'02.7"S 028°14'23.1"E	Females	October 2016
6	25°44'05.0"S 028°14'16.83"E	Females	October 2016
7	25°44'04.0"S 028°14'22.0"E	Females	October 2016
Yellow	25°44'02.8"S 28°14'27.2"E	Females	October 2018
Red	25°44'06.7"S 28°14'30.5"E	Females	October 2018
Blue	25°44'04.2"S 28°14'16.8"E	Females	October 2018
Green	25°44'04.6"S 28°14'30.5"E	Females	October 2018
