# Supplementary material for: Lifespan prolonging mechanisms and insulin upregulation without fat accumulation in long-lived reproductives of a higher termite
Source: Commun Biol. 2022 Jan 13;5:44. doi: 10.1038/s42003-021-02974-6 (PMC8758687; doi:10.1038/s42003-021-02974-6)
Supplement: Supplementary file 3 — Description of Additional Supplementary Files [file 42003_2021_2974_MOESM3_ESM.pdf]

Description of Additional Supplementary Files

File name: Supplementary Data 1
Description: GO terms significantly enriched in each of the WGCNA modules. Significance was calculated with a Fisher test within the R package topGO (Alexa, A. & Rahnenfuhrer, J. 2016) by comparing the frequency of GO terms within a module to their frequency within all genes.

File name: Supplementary Data 2
Description: Gene expression during queen maturation. Table representing standardized gene expression at each of the five queen stages (QT0-QT4). Number of replicates per group is provided in Supplementary Table 1. Annotations include WGCNA module (moduleColor) (Supplementary Fig. 2), gene name and gene acronyms in Drosophila melanogaster and Homo sapiens. Genes were considered significantly differentially expressed between adjacent queen
stage if the adjusted p-value (adj.P) was less than 0.05.

File name: Supplementary Data 3
Description: Normalised read counts for Macrotermes natalensis across 25 samples (IDs in column headers 2-26). The final two columns contain information on known orthologs in Drosophila melanogaster (Dmel) and Homo sapiens (Hsap). Reads were mapped to the genome with Hisat2 (Kim et al. 2019), counts were generated with htseq-count (Anders et al. 2019) and reads were normalised with DESeq2 (Love et al 2014) (see methods for more details).

File name: Supplementary Data 4
Description: Differential expression summary. Contains standardised expression per caste and queen stage, orthology information in Drosophila melanogaster and Homo sapiens, and the results of each pairwise differential expression analysis. For example, in the column "T2_K" a T2 signifies a significant upregulation in QT2 compared to kings, a "K" means an upregulation in kings, and "notDE" means no significant difference in expression. Differential expression was calculated with DESeq2 (Love et al 2014).

File name: Supplementary Data 5
Description: Contains new annotations of the two ILP genes reported in this study. These genes were annotated in the Macrotermes natalensis genome using EXONERATE (v2.2.0, Slater et al. 2005).

File name: Supplementary Data 6

Description: Contains protein sequences for the newly annotated ILPs, contained in supplementary data 5
