# Supplementary material for: Lifespan prolonging mechanisms and insulin upregulation without fat accumulation in long-lived reproductives of a higher termite
Source: Commun Biol. 2022 Jan 13;5:44. doi: 10.1038/s42003-021-02974-6 (PMC8758687; doi:10.1038/s42003-021-02974-6)
Supplement: Supplementary file 10 — Reporting Summary [file 42003_2021_2974_MOESM10_ESM.pdf]

nature research

Corresponding author(s): Mireille VASSEUR-COGNET

Last updated by author(s): November 30, 2021

Reporting Summary

Nature Research wishes to improve the reproducibility of the work that we publish. This form provides structure for consistency and transparency in reporting. For further information on Nature Research policies, see our Editorial Policies and the Editorial Policy Checklist.

Statistics

For all statistical analyses, confirm that the following items are present in the figure legend, table legend, main text, or Methods section.

n/a	Confirmed	
☐	☒	The exact sample size (*n*) for each experimental group/condition, given as a discrete number and unit of measurement
☐	☒	A statement on whether measurements were taken from distinct samples or whether the same sample was measured repeatedly
☐	☒	The statistical test(s) used AND whether they are one- or two-sided<br>*Only common tests should be described solely by name; describe more complex techniques in the Methods section.*
☐	☒	A description of all covariates tested
☐	☒	A description of any assumptions or corrections, such as tests of normality and adjustment for multiple comparisons
☐	☒	A full description of the statistical parameters including central tendency (e.g. means) or other basic estimates (e.g. regression coefficient) AND variation (e.g. standard deviation) or associated estimates of uncertainty (e.g. confidence intervals)
☐	☒	For null hypothesis testing, the test statistic (e.g. F, t, r) with confidence intervals, effect sizes, degrees of freedom and P value noted<br>*Give P values as exact values whenever suitable.*
☒	☐	For Bayesian analysis, information on the choice of priors and Markov chain Monte Carlo settings
☒	☐	For hierarchical and complex designs, identification of the appropriate level for tests and full reporting of outcomes
☒	☐	Estimates of effect sizes (e.g. Cohen's *d*, Pearson's *r*), indicating how they were calculated

Our web collection on statistics for biologists contains articles on many of the points above.

Software and code

Policy information about availability of computer code

Data collection	no software used
Data analysis	R (v. 3.6.1); R (v. 3.6.3) and R (v.4.0.2); R packages: [WGCNA (v. 1.69); topGO (v. 2.24.0); ComplexHeatmap (v. 2.1.0)] hisat2 (v. 2.1.0) ; htseq-count (v. 0.10.0); DESeq2 (v. 1.18.1); Cytoscape (v. 3.8.0); exonerate (v. 2.2.0); mafft (v. 7.397); dunn.test (v. 1.3.5); ggplot2 (v. 3.3.3); ggtern (v. 3.3.0)]; Metaboanalyst (v. 4.0); compositions (v. 2.0-1); vegan (v. 2.5.7); devtools (v.2.4.0); pairwiseAdonis (v.0.0.1), BLASTp (v.2.7.1+); t-coffee; iqtree2; iTOL (v.6);

For manuscripts utilizing custom algorithms or software that are central to the research but not yet described in published literature, software must be made available to editors and reviewers. We strongly encourage code deposition in a community repository (e.g. GitHub). See the Nature Research guidelines for submitting code & software for further information.

Data

Policy information about availability of data

All manuscripts must include a data availability statement. This statement should provide the following information, where applicable:

- Accession codes, unique identifiers, or web links for publicly available datasets
- A list of figures that have associated raw data
- A description of any restrictions on data availability

RNA-seq reads generated in this study have been deposited in Sequence Read Archive (BioProject ID: PRJNA685589 and BioSample accessions: SAMN17088123-SAMN17088147). The normalised expression data, differential expression results, ILP annotations and ILP sequences are available as supplementary data 3-6. Further data and scripts that support the findings of this study are available in Dryad with the identifier https://doi.org/10.5061/dryad.51c59zw7t. All other data are available from the corresponding author on reasonable request.

Field-specific reporting

Please select the one below that is the best fit for your research. If you are not sure, read the appropriate sections before making your selection.

☒ Life sciences ☐ Behavioural & social sciences ☐ Ecological, evolutionary & environmental sciences

For a reference copy of the document with all sections, see nature.com/documents/nr-reporting-summary-flat.pdf

Life sciences study design

All studies must disclose on these points even when the disclosure is negative.

Sample size	Sample size was determined upon availability of termite individuals
Data exclusions	No data were excluded.
Replication	Exact procedures and data analysis steps are provided to ensure reproducibility of the study.
Randomization	Termites were sampled at random among the available individuals of a given age or caste.
Blinding	The fat body transcriptomic /lipidomic analyses and the lipidomic /metabolomic analyses on hemolymph occurred with relabeled samples ensuring blinding.

Reporting for specific materials, systems and methods

We require information from authors about some types of materials, experimental systems and methods used in many studies. Here, indicate whether each material, system or method listed is relevant to your study. If you are not sure if a list item applies to your research, read the appropriate section before selecting a response.

Materials & experimental systems

n/a	Involved in the study	
☒	☐	Antibodies
☒	☐	Eukaryotic cell lines
☒	☐	Palaeontology and archaeology
☐	☒	Animals and other organisms
☒	☐	Human research participants
☒	☐	Clinical data
☒	☐	Dual use research of concern

Methods

n/a	Involved in the study	
☒	☐	ChIP-seq
☐	☒	Flow cytometry
☒	☐	MRI-based neuroimaging

Animals and other organisms

Policy information about studies involving animals; ARRIVE guidelines recommended for reporting animal research

Laboratory animals	Under our HFSP collaborative project, termite species Macrotermes natalensis imagoes were collected in 2016 and in 2018 during the spring swarming flights in Pretoria (South Africa) in an experimental field of the University of Pretoria (coordinates in Supplementary Table 8). Establishment of laboratory incipient colonies have been done for both field trips by following a protocol adapted from Lepage and Han & Bordereau (and Supplementary Fig. 8). The incipient colonies were kept in a breeding room with controlled conditions: 28°C, 85 % relative humidity with 12:12 photoperiod. Deionized water was provided in each box to keep the soil slightly but constantly moistened. Queens of laboratory colonies were sampled at 3 months of age (QT1), 9 months (QT2) and 31 months (QT3).
Wild animals	Macrotermes natalensis field colonies opened to provide animals in these experiments were followed over 20 years in Pretoria (South Africa) in an experimental field of the University of Pretoria (coordinates in Supplementary Table 8). Old female (small) workers (FW) and 20-years old queens (QT4) and kings (KT4) and male and female imagoes (QT0) were collected at swarming using nets and used to establish incipient colonies, all were sampled from the same colony (see Supplementary Table 1 for more details on sampling and replicates) .
Field-collected samples	Hemolymph were collected from individuals cold-anesthetized in cryotubes, quickly frozen in liquid nitrogen and kept at -80°C until use. Then, termites were killed by decapitation and abdominal fat body was collected and loaded in a tube containing RNAlater buffer (Invitrogen) and kept at -80°C until use. For lipids and metabolites analyses, the fat body was nitrogen-frozen crushed in a tube which was immediately frozen in liquid nitrogen and kept in -80°C until use. For ploidy analyses, the fat body was collected from one individual and loaded in a tube containing 200 µL of Cycletest PLUS DNA Reagent Kit buffer (Becton Dickinson) and kept in -80°C until use (see Supplementary Table 1 for more details on sampling and replicates).

Ethics oversight	Concerning insects no ethical approval was required. The study was conducted according to the Nagoya protocol. Samples (tissues and hemolymph) were exported at -80°C from Pretoria-South Africa to Bondy-France (permit 93010001).

Note that full information on the approval of the study protocol must also be provided in the manuscript.

Flow Cytometry

Plots

Confirm that:

- [ ] The axis labels state the marker and fluorochrome used (e.g. CD4-FITC).
- [ ] The axis scales are clearly visible. Include numbers along axes only for bottom left plot of group (a 'group' is an analysis of identical markers).
- [ ] All plots are contour plots with outliers or pseudocolor plots.
- [x] A numerical value for number of cells or percentage (with statistics) is provided.

Methodology

Sample preparation	Fat bodies (see Supplementary Table 1 for more details on sampling and replicates) were processed by Flow Cytometric Analysis with a Cycletest PLUS DNA Reagent Kit using propidium iodide (BD Biosciences, Le pont de Claix) as described by manufacturer's instructions. All procedures were adapted from Nozaki & Matsuura.
Instrument	Accuri C6 Flow Cytometer (BD Biosciences, Le Pont de Claix, France). Stained nuclei were analyzed at an excitation wavelength of 488 nm and a detector equipped with an 585/45 bandpass filter.
Software	We analysed the data using CFlow Plus (BD Biosciences, Le Pont de Claix, France).
Cell population abundance	Approximately 1,000 cell nuclei were acquired for each measurement.
Gating strategy	Debris were removed on an FSC-A/SSC-A dotplot and doublet were eliminated with and PI-FL2-H/ FL2-A dot plot. The nuclei were analyzed with a histogram PI-A. The 1C DNA peak was determined by the analysis of king's testis (sperm), allowing the identification of the 2C, 4C, and 8C peaks of the others samples.

- [x] Tick this box to confirm that a figure exemplifying the gating strategy is provided in the Supplementary Information.
